# Associative Learning from Verbal Action-Effect Instructions: A Replication and Investigation of Underlying Mechanisms

**DOI:** 10.5334/joc.284

**Published:** 2023-06-22

**Authors:** Yevhen Damanskyy, Torsten Martiny-Huenger, Elizabeth J. Parks-Stamm

**Affiliations:** 1 UiT The Arctic University of Norway, Tromsø, NO; 2 University of Southern Maine, USA

**Keywords:** Verbal instructions, Action-effect, Associative learning, Learning, Action-Control

## Abstract

According to the ideomotor principle, repeated experience with an action and its perceivable consequences (effects) establish action-effect associations. Research on verbal instructions indicates that such associations are also acquired from verbal information. In the present experiment (N = 651), first, we aimed to replicate unintentional response-priming effects from verbal action-effect instructions (direct replication; Condition 1). Second, we investigated the involvement of perceptual processes in the verbally induced response-priming effect by perceptually presenting (Condition 1) versus not presenting (Condition 2) the color that was subsequently named as an effect in the instructions. Third, we tested a saliency-based explanation of the verbally induced response-priming effect by highlighting all components (action and effect) without an association between them (Condition 3). Overall, we found the predicted response-priming effect following verbal action-effect instructions (overall conditions and in the replication Condition 1). Condition 2, which did not include perceptual information in the instructions, still showed a significant response-priming effect but was descriptively weaker compared to the effect of the replication Condition 1. Condition 3, which merely highlighted the action and effect component without endorsing an association, did not show a significant effect. In sum, our study provides further solid evidence that verbal instructions lead to unintentional response-priming effects. Other conclusions must be considered preliminary: The between-condition comparisons were descriptively in the predicted direction—perceptual aspects are relevant, and a saliency-based account can be excluded—but the differences in accuracy between conditions were not statistically significant.

According to the ideomotor principle, goal-directed actions are driven by anticipatory representations of their effects (i.e., action-effect; Elsner & Hommel, 2001; James & Hutchins, 1952). Our actions produce perceivable changes in the surroundings. The temporal overlap between actions and their perceptual effects results in the formation of associations between them. When a person mentally activates a particular perceptual effect (e.g., when forming an intention to achieve a certain outcome), it also activates the associated action that has led to the effect previously. This mechanism is postulated to enable goal-directed behavior.

Experimental procedures to test action-effect learning typically include two phases: learning and testing. In the learning phase, participants experience the co-occurrence of specific responses and their perceptual effects. The test phase is designed to evaluate the relations between those actions and their effects in choice-reaction tasks in which the previously-learned effect is encountered (Elsner & Hommel, 2001). The idea is that exposure to a learned effect should automatically activate the corresponding action. From a measurement perspective, this activation is inferred from an observed response bias in the choice-reaction task (compatibility effect).

We use the term “compatibility effects” (Kornblum et al., 1990) when responses in the test phase are facilitated or impeded by the presence of an effect stimulus from the learning phase. Perceiving the effect from the learning phase (as a target or a task-irrelevant stimulus) leads to a retrieval of a response that has become associated with that effect stimulus in the learning phase. When an associated response *matches* the required response in the test phase (compatible trials), responses are facilitated (i.e., shorter response times and/or fewer response errors). When the associated response is *different* from the required response (incompatible trials), responses are impeded (i.e., longer response times and more response errors).

There is ample evidence for compatibility effects resulting from action-effect learning based on direct experiences (Elsner & Hommel, 2001; Hommel, 2004; for a review see Shin et al., 2010; Waszak et al., 2012;). However, prior research also indicates that action-effect learning is not limited to learning from actual experience with an action-effect contingency. For example, Pfister et al. (2014) demonstrated that action-effect learning occurred in the absence of direct experiences with the action-effect pairing. While participants executed a response, they only imagined the anticipated outcome of their action. This was sufficient to produce response-compatibility effects that are indicative of action-effect learning.

Another study (Eder & Dignath, 2017) showed action-effect learning when both components were instructed before the test phase. However, participants directly experienced the action-effect contingencies in the test phase. While this may be interpreted as evidence for readily observable action-effect learning (i.e., early in the test phase) following instructed action-effect contingencies, the contribution of the instructions in Eder and Dignath (2017) are not clearly separable from the effect of learning from the first direct experiences in the test phase or from instruction/direct-experience interactions. Most relevant to our present focus are two recent publications that report studies with a clearer separation of instructions and direct experiences (Damanskyy et al., 2022; Theeuwes et al., 2015).

The experimental procedure in these two recent publications on verbal action-effect instructions (Damanskyy et al., 2022; Theeuwes et al., 2015) is similar to those that induce learning based on direct experiences. However, in the learning phase, instead of performing an action and perceiving the effect, participants see verbal instructions for specific action-effect relationships. The test phase is the same as in research from direct experiences. The influence of the verbal instructions on participants’ responses is tested in a categorization task where instruction-relevant features are visually presented to create response-instruction compatible and incompatible trials.

For example, Theeuwes et al. (2015) provided evidence that verbal action-effect instructions produce a compatibility effect that would be expected from learning based on direct experience. In three experiments, participants were provided with action-effect instructions (e.g., pressing the left key will remove the letter A from the grid filled with letters; “learning phase”). Before starting the task where these instructions should be applied (inducer task), participants completed a separate task that was unrelated to the instructions but contained features from them (diagnostic task/test phase). Participants were asked to judge whether the previously-encountered letters – including the letter from the action-effect instructions – were presented *upright* or *italic* by pressing the left or right key. Thus, instruction combinations for the diagnostic task were either compatible or incompatible with the action-effect instructions presented for the inducer task. The results showed a compatibility effect in the diagnostic task, pointing to an effect of the instructions on the subsequent performance.

Damanskyy et al. (2022) also provided evidence that action-effect instructions produce a compatibility effect, but with some conceptual changes. While the results of Theeuwes et al. (2015) can be explained by participants holding the action-effect instructions in working memory, the procedure of Damanskyy et al. made it less likely that the verbal instructions were held in working memory by creating a stronger separation between the action-effect instructions (learning phase) and the test phase. This was mainly achieved by testing the effects a few minutes after the instructions were presented (Damanskyy et al., 2022) rather than after a few seconds (Theeuwes et al., 2015). In the learning phase, participants memorized action-effect instructions (e.g., “To make the screen blue, I have to press the left key”). In the test phase, participants performed an ostensibly unrelated vowel-consonant categorization task. However, in some trials the background of the screen turned blue, creating response-instruction compatible and incompatible trials. The results showed a compatibility effect for the action-effect instructions on the participants’ response accuracy. This effect was observed despite the delay between the learning and test phase and the action-effect instructions never becoming relevant in the test phase. Thus, the authors concluded that in comparison to the study procedure by Theeuwes et al., it was less likely that participants held the action-effect instructions actively in working memory. This provides support for the idea that associations were formed while memorizing the action-effect instructions.

Two questions arise from this previous research. First, Damanskyy et al. (2022) involved not only verbal information in the instructions, but also a visual sample of the color blue that was referenced in the verbal instructions. This leads to questions about the contribution of visual perception in verbal action-effect learning. Does the mechanism underlying this verbally induced learning involve perceptual aspects? Second, an alternative explanation of the findings could be that the mere familiarity with the stimuli (effect and response), and not associative learning between them, could account for the findings (i.e., a saliency-based explanation). The present study was designed to address these two questions.

## Present Research

In the present research, we investigated three central aspects related to the effect of verbally induced action-effect instructions. First, we sought to provide a high-powered replication of an unintentional response-priming effect from a verbally processed – but never directly executed – action-effect contingency. Second, we investigated the relevance of perceptual processes for verbal action-effect learning. Third, we tested the idea that verbal action-effect instructions establish associative links against a saliency-based alternative explanation. The experimental procedure of this study was similar to Damanskyy et al. (2022). In the learning phase, participants read action-effect instructions formulated in an effect-action order (“To make the screen blue, I will press the [left/right] key”). In the test phase, participants categorized letters as a vowel or consonant by pressing the left or right key. On 1/4 of the trials, the screen background turned blue (i.e., action-effect; critical trials). Thus, the participants encountered the effect that was previously verbally linked to either a left or right response. Thus, the required categorization response (left or right) was either compatible or incompatible with the response specified in action-effect instructions.

Our study consisted of three between-participant conditions. The first condition (visual-verbal link) served as a standard for comparing the remaining two conditions and is an exact replication of Damanskyy et al. (2022; effect-action order). In this condition the presented verbal information included perception and action components that were combined to form an action-effect contingency (‘To make the screen blue, I will press the [left/right] key’). Before processing the verbal action-effect instruction (on a separate instruction page), participants were presented with the perceptual component (blue color) and told that this was the color referred to in later instructions.

The second condition (verbal link only) was identical to the first, except the blue color was not presented before the verbal instructions. This was designed to address the role of perceptual aspects in the link-formation process. If the presentation of the actual color before the verbal instructions influences the compatibility effect, this suggests that the learning mechanism that mediates the effect between reading the verbal instruction and observing a response-compatibility effect does not rely solely on language-like symbolic processes. Instead, perceptual processes would contribute to that mechanism.

The third condition (no verbal link) was designed to test the associative-learning account against a saliency-based alternative explanation. To that aim, the instruction presentation did not include the verbal action-effect contingency, but instead presented the effect and the action components independently from each other. On one page of the task instructions participants were informed that pressing a specific key (either right or left, counterbalanced by participant) was important and they should thus memorize the statement ‘I will press the [right/left] key.’ Later, on a different instruction page, participants were presented with the blue color sample and were informed that this color was relevant and will appear during the categorization task. In other words, we highlighted both components (perceptual and action aspects) but did not facilitate an association between them. If previous findings (i.e., Damanskyy et al., 2022) and those in the first two conditions are a result of associative learning and not merely a result of increased salience of the perceptual and action component, then we should observe a weaker effect in this third no verbal link (saliency-only) condition.

In sum, to provide further information about the mechanism involved in verbal action-effect learning, we compared three conditions. The “visual-verbal link” condition facilitated associative learning and provided exact information about the perceptual properties of the perceptual component. The “verbal link only” condition also facilitated associative learning between the instructed perceptual and action component but did not include the exact perceptual properties. The “no verbal link” condition highlighted the perceptual and action components but did not facilitate associative learning between them. We expected the strongest response-compatibility effect in the visual-verbal link condition, replicating Damanskyy et al. (2022). We expected a comparatively weaker response-compatibility effect in the verbal link only condition, and no effect in the no verbal link condition.

## Method

### Participants

A total of 655 English-speaking adults participated in the study (228 males, 417 females, and 10 missing responses). Participants’ age ranged from 18 to 50 (*M* = 32.1, *SD* = 8.8). The participants were recruited by the online participant recruitment platform Prolific and received monetary compensation. We removed four participants who participated in the study twice due to technical errors. The study was approved by the local ethics committee, and all participants provided informed consent. The required sample size to find the central response-compatibility effect was not calculated prior to the analysis but was instead based on prior experiences with a similar design (~200 participants; effect-action condition in Damanskyy et al., 2022, Exp. 2).

### Design

The study included three main conditions: *visual-verbal link, verbal link only, no verbal link*. In the instructions for the visual-verbal link condition participants saw an example of the critical stimulus (color blue) followed by the action-effect instructions. In the verbal link only condition, participants saw only the action-effect instructions. In the no verbal link condition, participants were presented separately with instructions for a specific response and an example of the to-be-presented color. The data collection of the no verbal link condition reported in this manuscript was done after the data collection of the other two conditions, once we realized that a design error (missing verbal-response factor) in the originally-collected third condition made it impossible to calculate a comparable response-compatibility effect. Because of this error and the subsequent changes, the relationship between the present research and the initial pre-registration (https://osf.io/qfmc6) is complicated. However, the overall hypotheses and technical details of the analyses (e.g., outlier exclusion) remain the same. Analyses were conducted only after data collection was completed for all conditions.

All three conditions included two within-subject factors (*required response* and *effect prime*) and one between-subject factor (*instructed response*). Required response represented a factor that specified what response was required from participants according to the categorization task instructions (i.e., left vs. right key). Effect prime specified whether the blue screen was present (critical) or absent (control) in a given trial. Instructed response was a between-participant factor indicating the instructed response in the action-effect instructions (i.e., “To make the screen blue, I will press the [left vs. right] key”) or response instructions (i.e., “I will press [left vs. right] key”). Key assignment to vowels/consonants was counterbalanced between participants.

### Procedure

The experiment was programmed using PsychoPy v. 2020.1.3 and uploaded to the Pavlovia server (Peirce et al., 2019; Pavlovia, 2021). Each participant received a link to the experiment allowing them to open it in the browser of their choice. Participants could not participate using devices other than a PC with a physical keyboard.

#### Learning phase

In the visual-verbal link condition, we presented an example of the critical stimulus (the color blue) and told them that this would be the color referred to later in the instructions. Afterwards, participants were presented with the action-effect instructions: e.g., “*To make the screen blue, I will press the right key*.” To consolidate the instruction in memory, the participants were told to repeat the action-effect sentence silently to themselves a few times. We informed participants that this instruction would become relevant in a later task. In the verbal link only condition participants saw only action-effect instructions; the critical color was not presented to them. They were also asked to repeat these instructions silently to themselves a few times. In the no verbal link condition participants saw an instruction that was not formulated in an action-effect manner and was not associated with the color blue (i.e., ‘I will press the right key’). They were also informed that this instruction was important and they were instructed to memorize the sentence. After some intermediate instructions, the participants were presented with the color blue and told that this color will appear in the subsequent categorization task. A few minutes passed between memorizing the critical action-effect instructions and starting the test-phase task. These minutes where filled with action-effect unrelated instructions (e.g., instructions how to perform the categorization task).

#### Test phase

The categorization task was identical for all conditions. The presented stimulus was either a vowel (A, O, or E) or a consonant (K, M, or T), and each appeared an equal number of times in random order. During this part, the participants judged whether a presented stimulus was a vowel by pressing the left key (A) or a consonant by pressing the right key (L). Along with each presented letter, the background color was either blue (effect prime present; 25% of the trials) or gray (effect prime absent; 75% of the trials). All stimulus-response combinations were equally distributed between the effect-prime present and effect-prime absent trials. We implemented a response deadline of 1500 ms. If a response was incorrect or longer than 1500 ms, an error feedback message was displayed for 1500 ms. Participants performed eight practice trials and 96 test trials. The practice trials did not include any critical trials (i.e., the background was always gray). Instructions for the test phase included information that the background color may change during the task and they were explicitly instructed to ignore these color changes and focus on the vowel-consonant categorization task.

### Data Preparation and Data Analysis

We used the R software package to prepare and analyze the data (R core Team, 2021). Response errors and reaction times were analyzed with a mixed ANOVA (*ez* package; Lawrence, 2016). In addition, the reaction time variable was log-transformed (Judd et al., 1995). No participant made excessively fast responses (i.e., more than 10% responses faster than 300 ms). Based on a boxplot outlier analysis (+/–3× interquartile range; Tukey 1977), we removed the data of 13 participants (>17% response errors). The final analyzed sample included 638 participants.

Responses with missed deadline were omitted (0.83%). Prior to the response time analysis, we removed all error responses (3.43%). No response was faster than 150 ms (i.e., fast guesses). In addition, we removed trials with response times beyond the mean +/– 3 times the standard deviation calculated by participant and within-participant conditions (1.11%).

We applied ANOVA with Type II Sums of Squares for statistical analysis as recommended for unbalanced groups and for models in which an interaction effect is of interest (Langsrud, 2003). Furthermore, we coded the three main conditions visual-verbal link, verbal link only, and no verbal link as ordered factors (i.e., 1, 2, 3 respectively) as we expected a successively weaker effect in each condition.

## Results

### Response error

The 3-way interaction effect between required response, effect prime, and instructed response for response errors was significant *F*(1, 633) = 14.1, *p* < .001, η_p_^2^ = .02. The 4-way interaction effect including the three main conditions did not reach the conventional significance level, *F*(1, 633) = 2.08, *p* = .149, η_p_^2^ < .01. The non-significant trend could indicate an effect in the opposite direction than predicted: for example, showing no replication of Damanskyy et al. (2022) in the direct replication (visual-verbal link) condition and the strongest effect in the alternative, saliency-based (no verbal link) condition. To investigate this possibility, we performed further analysis of each condition separately to test whether we replicate the previous results and whether the trend is in the predicted direction. All results of the ANOVA analysis with response errors as the dependent variable are presented in Appendix B. In the following subsections we report only the hypothesis-relevant effects. To simplify comparisons between the three conditions, we present the size of the compatibility effect for each condition at the end of this section (Figure 2).

#### Visual-verbal link (Replication of Damanskyy et al., 2022)

**Figure 1:**
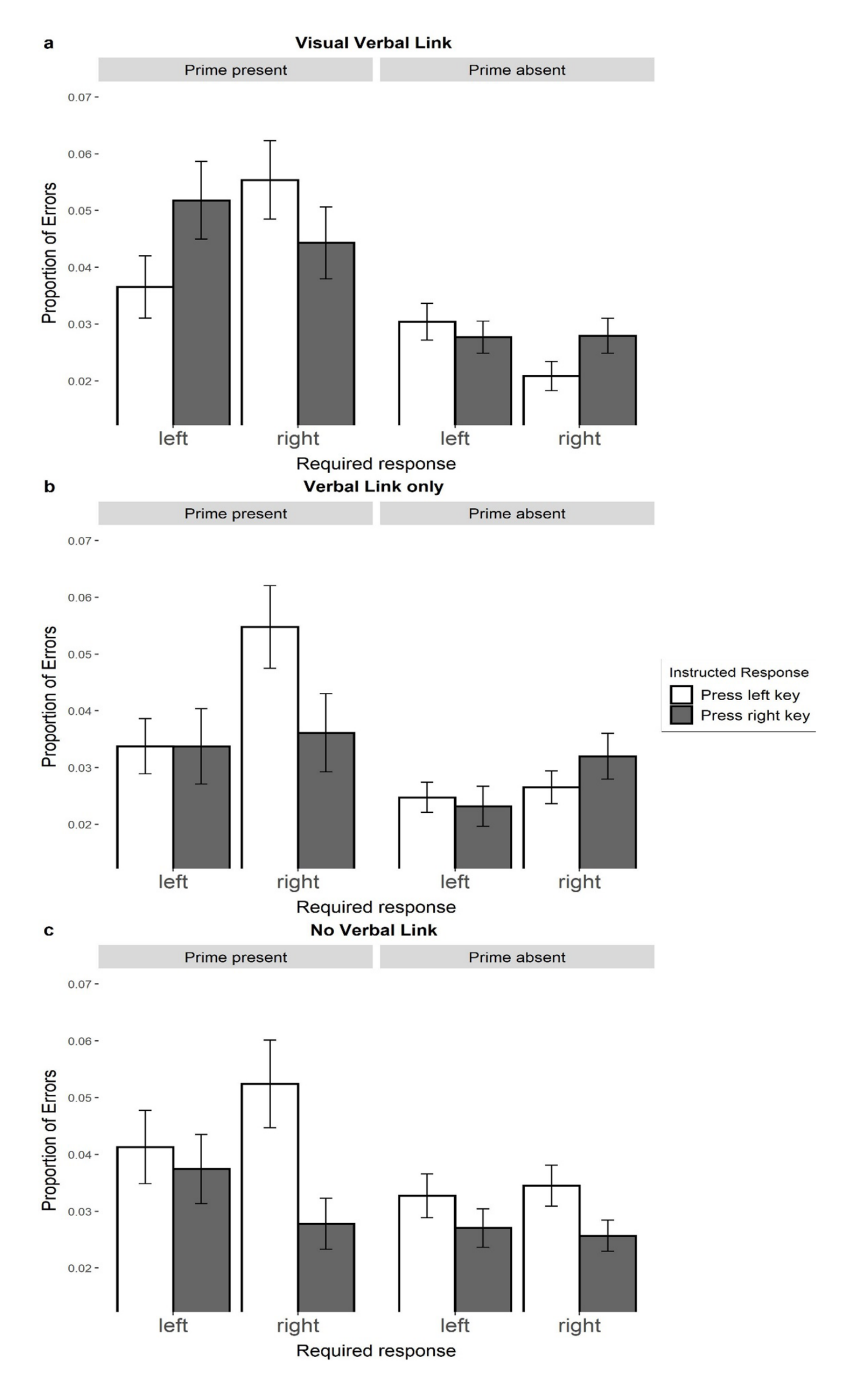
Mean response errors as a function of required response, effect prime, and instructed response for three conditions separately. The graph represents three different parts for three conditions separately: visual-verbal link **(a)**, verbal link only **(b)** no verbal link **(c)**. *Note*: Bars represent descriptive means with the standard errors for three main conditions. *Required response* specifies what response was required from participants in a given trial according to the categorization task instructions. *Effect prime* specifies whether the blue screen was present (critical) or absent (neutral) in a given trial. In the visual-verbal link (a) and verbal link only (b) conditions, *instructed response* indicates the instructed action formulated in action-effect manner (“To make the screen blue, I will press the left/right key”). In the no verbal link (c) condition the instructed action was a simple sentence (“I will press the left/right key”).

The expected 3-way interaction effect between required response, effect prime, and instructed response on response errors was significant *F*(1, 241) = 10.08, *p* = .002, η_p_^2^ = .04. We analyzed the experimental effect within prime present and prime absent trials separately. The expected 2-way interaction effect between required response and effect prime was significant for the trials with the effect prime present *F*(1, 241) = 7.36, *p* = .007, η_p_^2^ = .02. The same 2-way interaction effect was marginally significant for the trials with the effect prime absent *F*(1, 241) = 3.31, *p* = .070, η_p_^2^ = .01. The visual inspection of both 2-way interactions (Figure 1a) illustrate that the experimental effect within prime present trials was in the expected direction (compatible instructed and required responses are facilitated), whereas the effect in prime absent trials contained a tendency of the reverse pattern.

**Figure 2:**
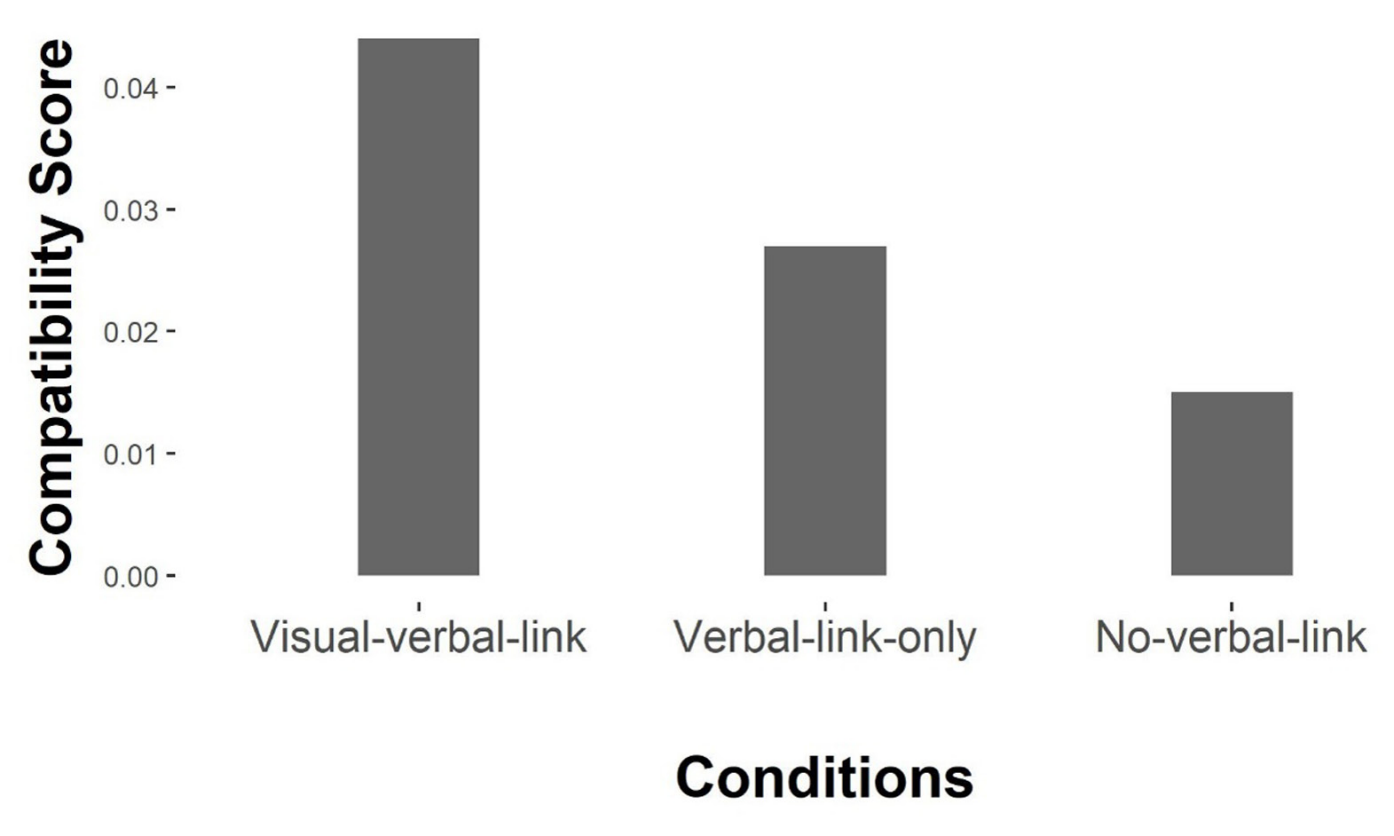
Compatibility gain scores of response errors. *Note*: Bar represents descriptive mean compatibility gain scores of response errors for each condition.

#### Verbal link only

The 3-way interaction effect between required response, effect prime, and instructed response was significant *F*(1, 201) = 3.98, *p* = .047, η_p_^2^ = .01. We analyzed the data further separately for the prime present and prime absent trials. For the critical prime-present trials, the 2-way interaction effect was not significant *F*(1, 201) = 1.89, *p* = .171, η_p_^2^ = .00. Similarly, the same interaction effect was also not significant for prime-absent control trials *F*(1, 201) = 2.41, *p* = .122, η_p_^2^ = .01. Visual inspection of the result pattern (Figure 1b) nonetheless indicates a pattern in the expected direction. Whereas the prime absent trials (left pane) indicate facilitation of responses in which instructed and required response are compatible, this pattern is reversed in the prime-absent trials (right pane).

#### No verbal link

In contrast to the previous two conditions, the 3-way interaction effect between required response, effect prime and instructed response was not significant *F*(1, 189) = 1.13, *p* = .289, η_p_^2^ = .00. The separate analyses for the prime present *F*(1, 189) = 2.07, *p* = .152, η_p_^2^ = .00 and prime absent trials *F*(1, 189) = 0.24, *p* = .622, η_p_^2^ = .00 also did not show significant effects. Figure 1c illustrates the visual presentation of this analysis.

#### Response compatibility score

As the magnitude of each compatibility effect is not easily visible from the three 3-way interaction effects illustrated in Figure 1, we calculated a response compatibility score for each experimental condition. The score is calculated by the sum of both compatibility effects in the critical prime present condition, minus the same sum calculated for the control condition (prime absent). The subtraction of the control condition “compatibility” effect results in an overall zero score (i.e., no effect) if the control condition shows the same result pattern as the critical experimental condition. The more positive the compatibility score, the larger the observed facilitation effect of compatible configurations and/or interference from incompatible configurations in the critical (prime present) condition as compared to the control (no prime) condition. Appendix A presents an example of this calculation procedure.

As illustrated in Figure 2, although the overall 4-way interaction effect is not significant (*p* = .149), the result pattern is descriptively in the predicted direction. The visual-verbal link condition shows the strongest compatibility effect, and the effect in the verbal link only condition is weaker. The no verbal link condition shows the weakest compatibility effect.

### Reaction Time

The 3-way interaction effect between required response, effect prime, and instructed response was not significant *F*(1, 633) = 1.24, *p* = .266, η_p_^2^ = .00. The 4-way interaction effect including the three main conditions was also not significant *F*(1, 633) = 0.23, *p* = .635, η_p_^2^ = .00. To stay consistent with the response errors analysis and to evaluate potential speed-accuracy trade-offs, we analyzed each condition separately. None of the 3-way or 2-way interaction effects are significant (all ps > .201). Appendix C presents the full ANOVA result tables for the reaction times analysis.

#### Visual-verbal link

The 3-way interaction between required response, prime, and instruction response was not significant *F*(1, 241) = 1.03, *p* = .310, η_p_^2^ < .01. The analysis revealed a significant main effect of prime, indicating that participants responded slower on critical trials than on control trials. We analyzed further the experimental effect within effect prime present (critical) and effect prime absent (control) trials separately to evaluate potential influence of speed accuracy trade-off. The 2-way interaction between required response and effect prime in critical trials was not significant *F*(1, 241) = 0.05, *p* = .831, η_p_^2^ < .01. The same 2-way interaction was also not significant within control trials *F*(1, 241) = 1.61, *p* = .206, η_p_^2^ < .01. These results indicate the speed-accuracy trade-off did not affect participants’ response errors.

#### Verbal link only

The 3-way interaction between required response, prime, and instructed response was not significant *F*(1, 201) = 0.49, *p* = .483, η_p_^2^ < .01. We analyzed further the experimental effect within effect prime present (critical) and effect prime absent (control) trials separately to evaluate the potential influence of a speed-accuracy trade-off. The 2-way interaction was not significant in both critical trials *F*(1, 201) = 0.09, *p* = .765, η_p_^2^ < .01 and control trials *F*(1, 201) = 0.48, *p* = .487, η_p_^2^ < .01.

#### No verbal link

The 3-way interaction between required response, prime, and instructed response was not significant *F*(1, 189) = 0.12, *p* = .735, η_p_^2^ < .01. We analyzed further the experimental effect within effect prime present (critical) and effect prime absent (control) trials separately to evaluate the potential influence of a speed-accuracy trade-off. The 2-way interaction was not significant in both critical trials *F*(1, 189) = 1.88, *p* = .172, η_p_^2^ < .01 and control trials *F*(1, 189) = 2.30, *p* = .131, η_p_^2^ < .01.

#### Response compatibility score

The compatibility scores of response times were calculated in the same ways as the compatibility scores of response errors.

## Discussion

The aim of the present study was to provide a further test of verbally induced response-compatibility effects and to investigate some of the underlying conditions. We will start by discussing the visual-verbal ink condition as it is an exact replication of previous results that we included as a baseline against which to compare the outcomes of the other conditions.

### Verbal Association and Color Specification (Visual-Verbal Link)

The results from the visual-verbal link condition replicated findings to the study of Damanskyy et al. (2022; effect-action order). Based on the response error analysis, the action-effect instructions influenced participants’ accuracy in the prime-present trials compared to the prime-absent control trials. Specifically, participants made fewer errors in compatible trials (i.e., when the required response matched the instructed response from the unrelated action-effect instructions), and more errors in incompatible trials (i.e., a compatibility effect). In the prime-absent trials, this compatibility effect was not observed. The response time analysis of this condition did not indicate a speed-accuracy trade-off. As an intermediate conclusion, the visual-verbal link condition provides a high-powered replication of previous findings (Damanskyy et al., 2022). They indicate that verbal action-effect instructions lead to unintentional response priming effects even when the instruction phase and the test phase are separated by a few minutes and the action-effect instructions are never used during the test phase. These results parallel research on the unintentional influences of stimulus-response instructions (e.g., Liefooghe & De Houwer, 2018).

### Verbal Association without an Associated Visual Cue (Verbal Link Only)

The verbal link only condition aimed to evaluate the role of a perceptual component in the effect of verbal instructions on action. In this condition participants did not see the example of the blue color patch before processing the action-effect instructions. The analysis still revealed a significant compatibility effect in the expected direction. As in the visual-verbal link condition, participants made fewer errors in prime-present trials when the required response matched the instructed response from the action-effect instructions, and the response time analyses did not indicate a speed-accuracy trade-off. By itself, this condition that includes only a small design change provides another replication of unintentional response-priming effects from verbal instructions.

**Figure 3:**
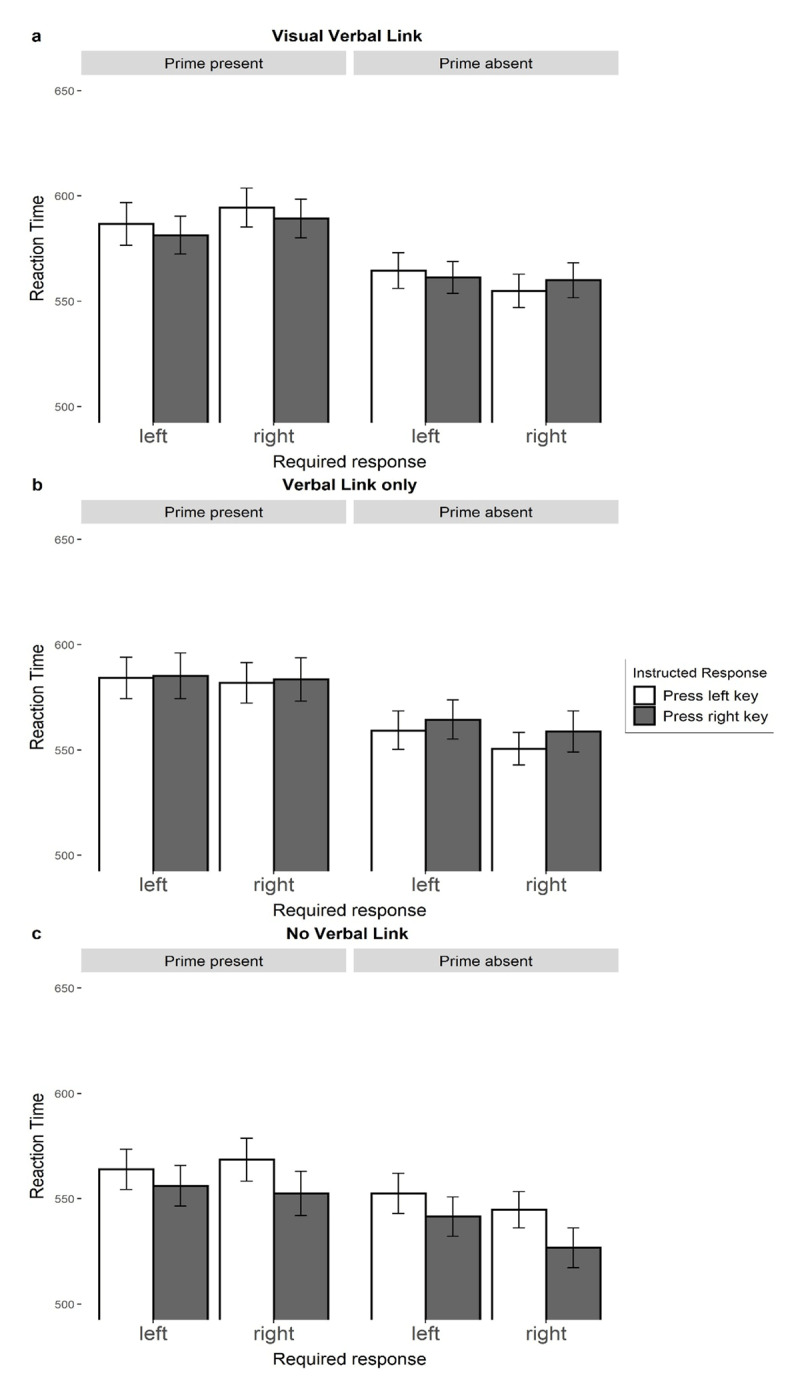
Mean response times as a function of required response, effect prime, and instructed response for three conditions separately. The graph represents three different parts for three conditions separately: visual-verbal link **(a)**, verbal link only **(b)** no verbal link **(c)**. *Note*: Bars represent descriptive means with the standard errors for three main conditions. *Required response* specifies what response was required from participants in a given trial according to the categorization task instructions. *Effect prime* specifies whether the blue screen was present (critical) or absent (neutral) in a given trial. In the visual-verbal link (a) and verbal link only (b) conditions, *instructed response* indicates the instructed action formulated in action-effect manner (“To make the screen blue, I will press the left/right key”). In the no verbal link (c) condition the instructed action was a simple sentence (“I will press the left/right key”).

Descriptively, the compatibility effect in this condition was smaller than in the visual-verbal link condition (see Figures 2 & 3 for summaries of the compatibility effect scores). The only methodological difference was that participants in the visual-verbal link condition were exposed to a more exact specification of the color represented the action-effect instructions than those in the verbal link only condition. If the mechanism from verbal instructions to responses was based only on abstract, symbolic representations of the information, visually perceiving the color during the instruction phase would not have made a difference for the resulting compatibility effect. While we only provide descriptive evidence that the color presentation made a difference, similar results have also been previously reported by Schmidt and Zelinsky (2009) and Wolfe et al. (2004), using a visual search paradigm. This research demonstrated that visual search for a stimulus is not as effective when it is only presented in an abstract form (compared to conditions in which the priming stimulus was visually presented along with a verbal specification).

### No Verbal Association (No Verbal Link)

The no verbal link condition was included to evaluate whether the effect of action-effect instructions is based on associative learning, or if the results could be explained by a saliency-based alternative account. If the compatibility effect indeed stems from an associative link between the stimulus and response components and not merely familiarity with these components and an independent temporary activation of both components, then presenting them separately from each other (i.e., without linking them) should result in a weaker (or absent) compatibility effect. For the first time in a series of five tests (Damanskyy et al., 2022, Exp. 1 & 2 and the first two conditions of the present study), we predicted no effect and found no significant compatibility effect—the pattern of response errors did not differ between the prime-present and prime-absent trials. Although we certainly hoped for a clearer outcome regarding the statistical analyses between the different conditions, we want to emphasize the importance of such control conditions. Independent of whether effects of verbal instructions are attributed to associative learning or alternative proposals about the components’ relationships (e.g., propositional learning; Sun et al., 2020), there is always the possibility that “mere exposure” to the instruction components, independent of their instructed relationships, can influence subsequent responses. Thus, experimental designs should account for such possibilities as we did in our design, even if it increases the likelihood of less clear-cut statistical outcomes.

### Limitations

The central limitation of the present research is in the non-significant 4-way interaction effect. Thus, while we have evidence that the instructions affected subsequent responses (3-way interaction effect), our conclusions related to differences between the conditions should be considered in relation to related research and as a starting point to continuously putting them to the test. We nonetheless presented the descriptive condition differences as they corresponded to our hypotheses. We expected the strongest compatibility effect for the visual-verbal link condition, and a comparatively weaker effect in the verbal link only condition. Furthermore, we expected and found the weakest compatibility effect in the no verbal link condition.

Overall, the present findings are based on the analysis of response errors. The analysis of response times serves to rule out a speed-accuracy trade-off as an explanation of the findings, mirroring the pattern found by Damanskyy et al. (2022). Mekawi and Bresin (2015) suggest that short deadlines (i.e., instructing participants to emphasize speed over accuracy) reduce variability within response times, which in turn increase error rates—leading to greater probability of finding an experimental effect within response errors. Researchers should consider using experimental designs without a response deadline to show more variability between conditions in response times. This, however, will create the potential for speed-accuracy trade-offs, which could complicate the analyses.

**Figure 4:**
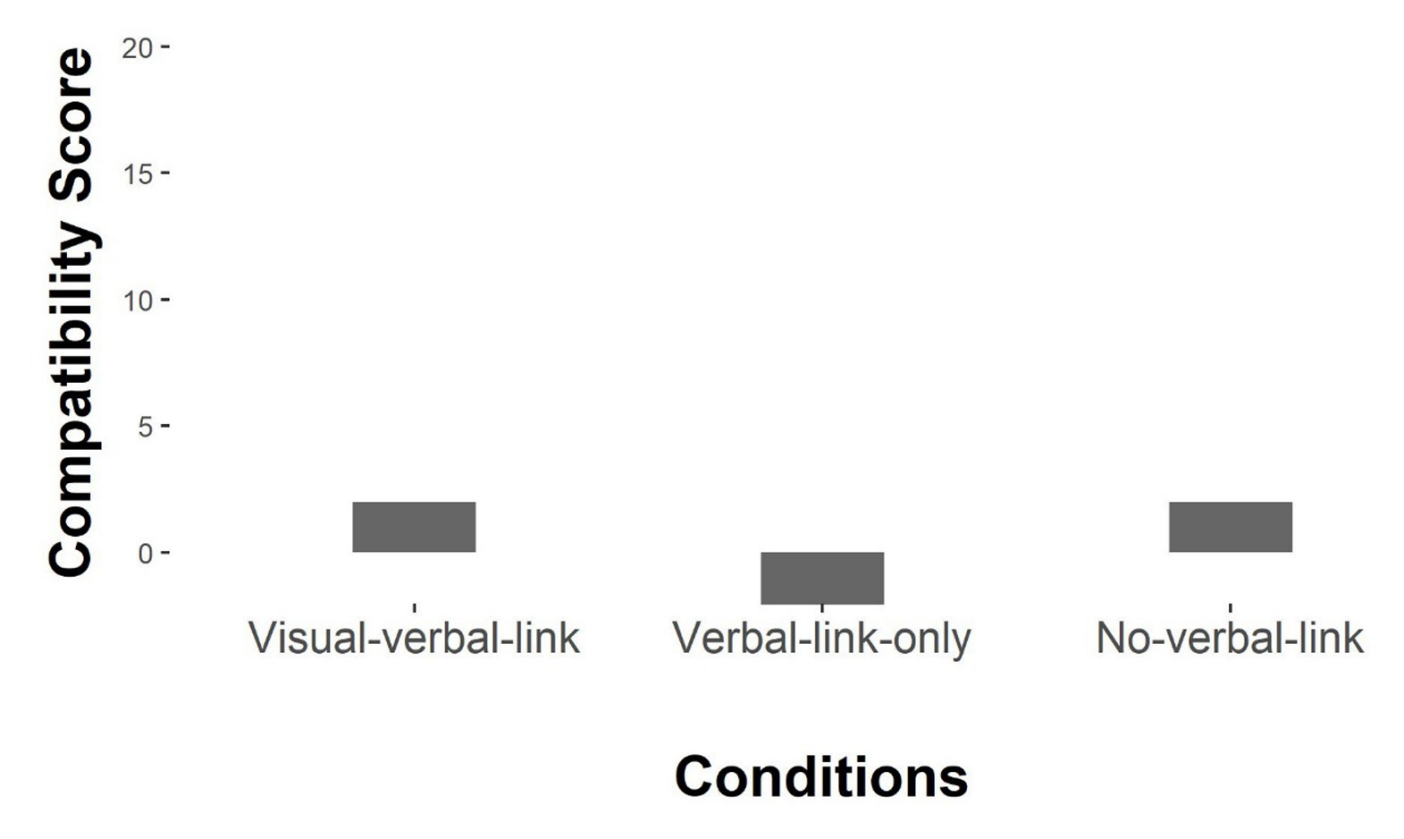
Compatibility gain scores of response times. *Note*: Bar represents descriptive mean compatibility gain scores of response errors for each condition.

The descriptive pattern of response errors and response times shown in Figure 1 and Figure 4 suggests that participants responded more slowly and made more errors in the prime-present trials (blue background) compared to the prime-absent trials (gray background). However, since the sudden background color change may have negatively impacted participants’ performance in the prime-present trials, the absolute difference between critical and control trials cannot be accurately estimated without an additional control condition. Since our prime-present trials involve both a verbal priming effect and the potential interference effect of a sudden background change, various combinations of interference are possible, including facilitation, facilitation and interference, or only interference. Therefore, to separate and differentiate the possible effect of the sudden background-color change, future studies should include an additional control condition that includes a background-color change without a verbal link between that color and response. Although our present data cannot differentiate between these possibilities, this limitation does not diminish the informative value of the observed interaction effect that suggests that verbal priming systematically influenced the responses.

## Conclusion

The present study provides another illustration of a verbally-induced response-compatibility effect. Unintentional response-priming effects are well documented (e.g., Shin et al., 2010), but typically derive from associations that are well-learned from direct experiences. In the present study, we observed response-priming effects following the memorization of a verbal representation of the action-effect contingency without any prior direct experiences with that contingency.

Beyond this central effect, we provide some initial evidence that perceptual aspects play a role in the mechanism that mediates the effect of verbal information on subsequent responses, and we find additional support for an associative-learning mechanism from verbal information to action by providing evidence against an explanation based solely on familiarity (or salience) of the individual perception and action components. These findings are in line with previously-presented theoretical perspectives (Martiny-Huenger et al., 2015, 2017) that suggest verbal information induces experiential simulations (e.g., Barsalou, 1999) that can then lead to (associative) learning that is similar to learning from direct experiences.

## Data Accessibility Statement

The datasets generated during the current study are available via the Open Science Framework (OSF): https://osf.io/28m6u/?view_only=fb3e8821628e41f1bf688ae71118b873.

The R script and R output are also available vis OSF: https://osf.io/28m6u/?view_only=fb3e8821628e41f1bf688ae71118b873.

The pre-registered hypothesis is also available via OSF: https://osf.io/qfmc6.

## Additional File

The additional file for this article can be found as follows:

10.5334/joc.284.s1 Appendices. Appendice A, B to C.
