# Supplementary material for: Associative Learning from Verbal Action-Effect Instructions: A Replication and Investigation of Underlying Mechanisms
Source: J Cogn. 2023 Jun 22;6(1):28. doi: 10.5334/joc.284 (PMC10289050; doi:10.5334/joc.284)
Supplement: Appendices. — Appendice A, B to C. [file joc-6-1-284-s1.pdf]

Appendix A

In this example we provide an example how we calculated the compatibility scores. We will only present a calculation procedure for the compatible scores in the visual-verbal link condition to avoid unnecessary repetition. The procedure for verbal-link only and no-verbal link conditions is the same and corresponding descriptive means can be found in the R output file (item 3.3) at https://osf.io/28m6u/?view_only=fb3e8821628e41f1bf688ae71118b873.

Table B1

Descriptive Statistics of the Response Errors in Visual-Verbal link condition

Required Response	Trial Type	Sentence Direction	Response Errors (%)	
			M	*SD*
Left	Critical	Press Left	.038	.059
Left	Critical	Press Right	.059	.088
Left	Control	Press Left	.031	.035
Left	Control	Press Right	.029	.034
Right	Critical	Press Left	.058	.074
Right	Critical	Press Right	.047	.072
Right	Control	Press Left	.023	.032
Right	Control	Press Right	.032	.043

The formula we used to calculate the compatible scores is the following:

**Step**	**Description**	**Formula**
1	Calculate critical response difference	CrD = (CrL.incompatible - CrL.compatible) + (CrR.incompatible - CrR.compatible)
2	Calculate control response difference	CnD = (CnL.incompatible - CnL.compatible) + (CnR.incompatible - CnR.compatible)
3	Calculate compatibility score	Compatibility Score = CrD - CnD

where:

- CrL: Critical left response
- CrR: Critical right response
- CnL: Control left response
- CnR: Control right response
- CrD: Critical response difference
- CnD: Control response difference

Calculations:

Visual-verbal link condition

((.059-.038) + (.058-.047)) – ((.029-.031) + (.023 - .032)) = .044

Verbal link only condition

((.038 - .036) + (.057 - .040)) – ((.025 - .028) + (.030 - .036)) = .027

No verbal link condition

((.038 - .048) + (.056 - .028)) - ((.027 - .037) + (.038 - .025)) = .015

The final numbers are overall compatibility scores that are illustrated on Figure 2.

Appendix B

Table B1. *Anova results for response errors*

Predictor	df_{Num}	df_{Den}	SS_{Num}	SS_{Den}	F	p	η^2_g
(Intercept)	1	633	3.76	2.96	804.56	.000	.32
Instructed response	1	633	0.01	2.96	1.94	.164	.00
Main condition	1	633	0.00	2.96	0.85	.356	.00
Required response	1	633	0.01	1.65	2.00	.157	.00
Effect prime	1	633	0.16	1.67	59.48	.000	.02
Instructed response × main condition	1	633	0.04	2.96	8.71	.003	.01
Instructed response × required response	1	633	0.01	1.65	5.03	.025	.00
Main condition × required response	1	633	0.00	1.65	0.02	.890	.00
Instructed response × effect prime	1	633	0.00	1.67	1.07	.301	.00
Main condition × effect prime	1	633	0.01	1.67	5.25	.022	.00
Required response × effect prime	1	633	0.00	1.62	1.12	.291	.00
Instructed response × main condition × required response	1	633	0.00	1.65	0.00	.978	.00
Instructed response × main condition × effect prime	1	633	0.00	1.67	0.96	.327	.00
Instructed response	1	633	0.04	1.62	14.07	.000	.00

× required response × effect prime							
Main condition × required response × effect prime	1	633	0.00	1.62	0.44	.510	.00
Instructed response × main condition × required response × effect prime	1	633	0.01	1.62	2.08	.149	.00

Note. df_{Num} indicates degrees of freedom numerator. df_{Den} indicates degrees of freedom denominator. SS_{Num} indicates sum of squares numerator. SS_{Den} indicates sum of squares denominator. η^2_g indicates generalized eta-squared.

Table B2. *Anova results for response errors (Visual – Verbal link condition)*

Predictor	df_{Num}	df_{Den}	SS_{Num}	SS_{Den}	F	p	η^2_g
(Intercept)	1	241	1.58	1.26	303.01	.000	.32
Instructed response	1	241	0.00	1.26	0.86	.354	.00
Required response	1	241	0.00	0.61	0.02	.892	.00
Effect prime	1	241	0.11	0.80	33.62	.000	.03
Instructed response × required response	1	241	0.01	0.61	2.54	.112	.00
Instructed response × effect prime	1	241	0.00	0.80	0.05	.827	.00
Required response × effect prime	1	241	0.00	0.67	0.62	.431	.00
Instructed response × required response × effect prime	1	241	0.03	0.67	10.08	.002	.01

Note. df_{Num} indicates degrees of freedom numerator. df_{Den} indicates degrees of freedom denominator. SS_{Num} indicates sum of squares numerator. SS_{Den} indicates sum of squares denominator. η^2_g indicates generalized eta-squared.

Table B3. *Anova results for response errors (Verbal link only condition)*

Predictor	df_{Num}	df_{Den}	SS_{Num}	SS_{Den}	F	p	η^2_g
(Intercept)	1	201	1.11	0.90	248.67	.000	.31
Instructed response	1	201	0.00	0.90	0.43	.511	.00
Required response	1	201	0.02	0.57	5.92	.016	.01
Effect prime	1	201	0.04	0.48	16.24	.000	.02
Instructed response × required response	1	201	0.00	0.57	0.26	.611	.00
Instructed response × effect prime	1	201	0.00	0.48	1.54	.215	.00
Required response × effect prime	1	201	0.00	0.49	1.11	.293	.00
Instructed response × required response × effect prime	1	201	0.01	0.49	3.98	.047	.00

Note. df_{Num} indicates degrees of freedom numerator. df_{Den} indicates degrees of freedom denominator. SS_{Num} indicates sum of squares numerator. SS_{Den} indicates sum of squares denominator. η^2_g indicates generalized eta-squared.

Table B4. *Anova results for response errors (No verbal link condition)*

Predictor	df_{Num}	df_{Den}	SS_{Num}	SS_{Den}	F	p	η^2_g
(Intercept)	1	189	1.08	0.80	253.89	.000	.34
Instructed response	1	189	0.04	0.80	10.56	.001	.02
Required response	1	189	0.00	0.46	0.03	.853	.00
Effect prime	1	189	0.02	0.39	10.03	.002	.01
Instructed response × required response	1	189	0.01	0.46	2.26	.134	.00
Instructed response × effect prime	1	189	0.00	0.39	1.37	.243	.00
Required response × effect prime	1	189	0.00	0.45	0.00	.945	.00
Instructed response × required response × effect prime	1	189	0.00	0.45	1.13	.289	.00

Appendix C

Table C1. *Anova results for response times*

Predictor	df_{Num}	df_{Den}	SS_{Num}	SS_{Den}	F	p	η^2_g
(Intercept)	1	633	822461145.62	20520020.06	25371	.000	.97
Instructed response	1	633	9443.62	20520020.06	0.29	.590	.00
Main condition	1	633	282535.22	20520020.06	8.72	.003	.01
Required response	1	633	3670.62	1195321.88	1.94	.164	.00
Effect prime	1	633	399185.99	837135.89	301.84	.000	.02
Instructed response × main condition	1	633	18441.15	20520020.06	0.57	.451	.00
Instructed response × required response	1	633	345.33	1195321.88	0.18	.669	.00
Main condition × required response	1	633	4022.96	1195321.88	2.13	.145	.00
Instructed response × effect prime	1	633	1642.09	837135.89	1.24	.266	.00
Main condition × effect prime	1	633	9074.34	837135.89	6.86	.009	.00
Required response × effect prime	1	633	16730.56	663080.69	15.97	.000	.00
Instructed response × main condition ×	1	633	3893.80	1195321.88	2.06	.152	.00

required response							
Instructed response × main condition × effect prime	1	633	2283.20	837135.89	1.73	.189	.00
Instructed response × required response × effect prime	1	633	1299.95	663080.69	1.24	.266	.00
Main condition × required response × effect prime	1	633	194.62	663080.69	0.19	.667	.00
Instructed response × main condition × required response × effect prime	1	633	235.80	663080.69	0.23	.635	.00

Table C2. *Anova results for response times (Visual Verbal link condition)*

Predictor	df_{Num}	df_{Den}	SS_{Num}	SS_{Den}	F	p	η^2_g
(Intercept)	1	241	325379537.74	8174294.33	9593.06	.000	.97
Instructed response	1	241	833.98	8174294.33	0.02	.876	.00
Required response	1	241	615.51	545825.32	0.27	.603	.00
Effect prime	1	241	202335.20	355461.64	137.18	.000	.02
Instructed response × required response	1	241	379.79	545825.32	0.17	.683	.00
Instructed response × effect prime	1	241	2497.35	355461.64	1.69	.194	.00
Required response × effect prime	1	241	11209.16	280217.67	9.64	.002	.00
Instructed response × required response × effect prime	1	241	1202.59	280217.67	1.03	.310	.00

Table C3. *Anova results for response times (Verbal link only condition)*

Predictor	df_{Num}	df_{Den}	SS_{Num}	SS_{Den}	F	p	η^2_g
(Intercept)	1	201	263985820	6205901.20	8550.11	.000	.97
Instructed response	1	201	8839.53	6205901.20	0.29	.593	.00
Required response	1	201	6204.89	360723.92	3.46	.064	.00
Effect prime	1	201	132036.67	307625.61	86.27	.000	.02
Instructed response × required response	1	201	16.71	360723.92	0.01	.923	.00
Instructed response × effect prime	1	201	1283.53	307625.61	0.84	.361	.00
Required response × effect prime	1	201	1230.13	226451.79	1.09	.297	.00
Instructed response × required response × effect prime	1	201	557.53	226451.79	0.49	.483	.00

Table C4. *Anova results for response times (No verbal link condition)*

Predictor	df_{Num}	df_{Den}	SS_{Num}	SS_{Den}	F	p	η^2_g
(Intercept)	1	189	233387134.79	6096564.68	7235.25	.000	.97
Instructed response	1	189	50121.42	6096564.68	1.55	.214	.01
Required response	1	189	3296.88	285542.59	2.18	.141	.00
Effect prime	1	189	73902.77	173536.87	80.49	.000	.01
Instructed response × required response	1	189	4589.44	285542.59	3.04	.083	.00
Instructed response × effect prime	1	189	444.37	173536.87	0.48	.487	.00
Required response × effect prime	1	189	6502.53	154050.67	7.98	.005	.00
Instructed response × required response × effect prime	1	189	93.88	154050.67	0.12	.735	.00
